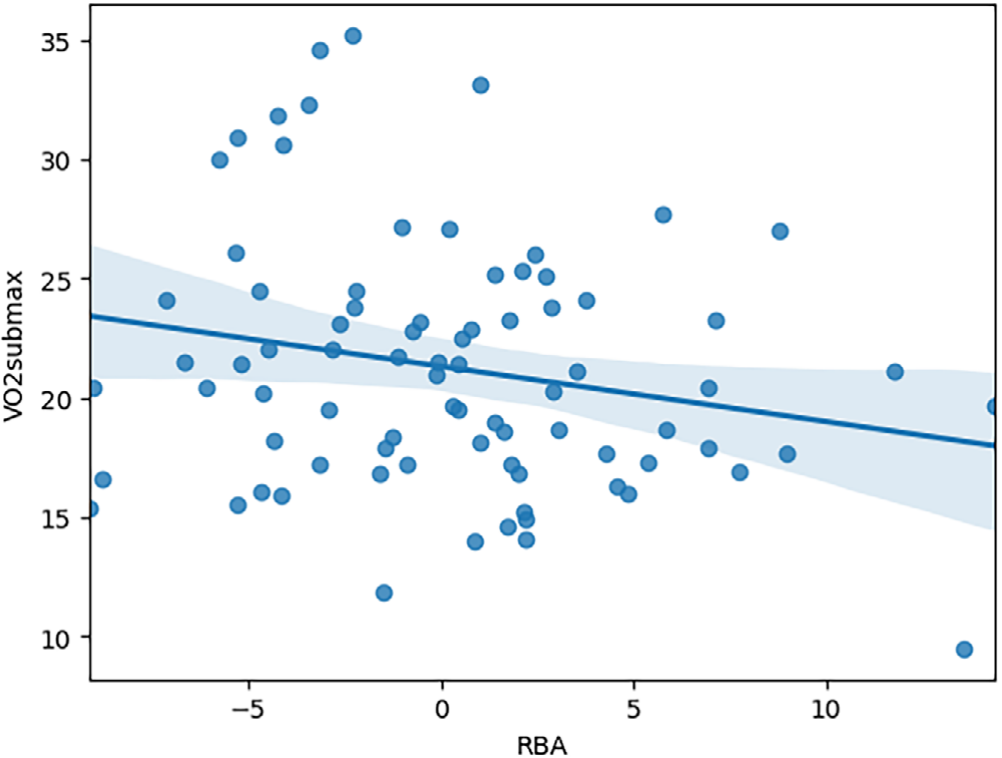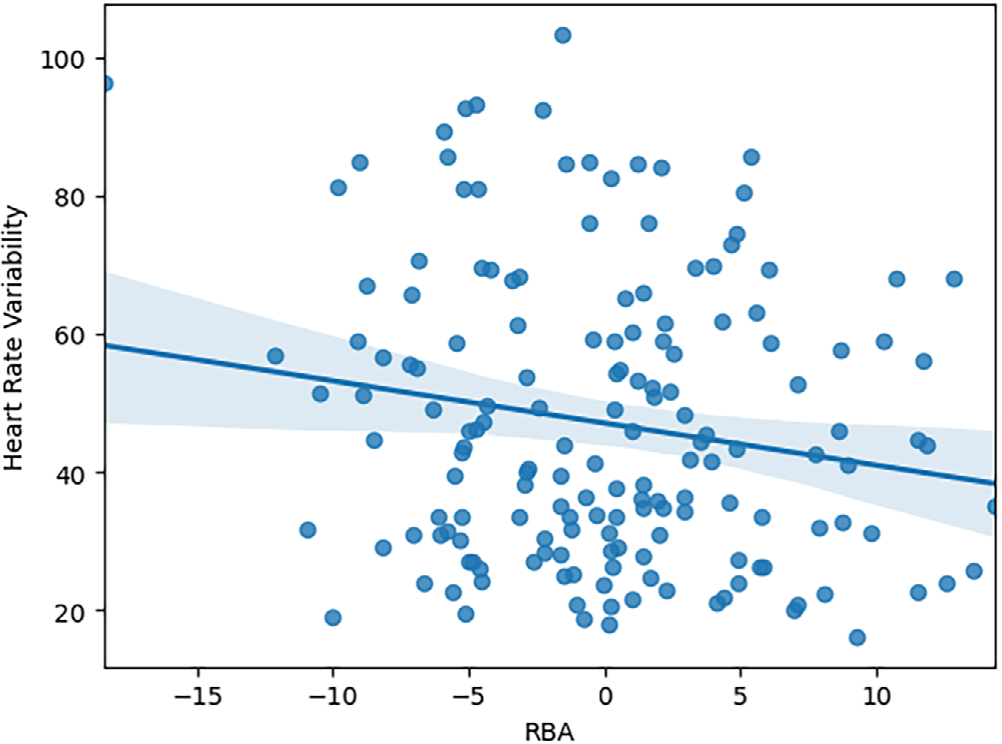# Heart rate variability and cardiorespiratory fitness associated with biological brain aging in a healthy adult lifespan sample

**DOI:** 10.1002/alz.095260

**Published:** 2025-01-09

**Authors:** Yunglin Gazes, Anna Mackay‐Brandt, Stan Colcombe

**Affiliations:** ^1^ Nathan Kline Institute, Orangeburg, NY USA

## Abstract

**Background:**

Measures of cardiovascular health have long been associated with brain health. For example, aerobic capacity (VO2max) is associated with preservation of functional and structural brain integrity in later life. Recent work has highlighted the utility of relative brain age (RBA; the age‐corrected difference between a brain’s chronological and estimated biological ages) to examine the rate of biological brain aging. However, the impact of cardiovascular factors on RBA remains underexplored. Here, we examine estimates of biological brain aging (via RBA) in the context of two key measures of cardiovascular health: aerobic fitness (VO2max), and heart rate variability (HRV) ‐ a measure of autonomic regulation associated with cognitive control, emotional regulation, and risk of neurological conditions, including dementia.

**Method:**

Data were obtained from ‘Mapping Interindividual Variation in the Aging Connectome’, an open‐science, community‐ascertained neuroimaging study with deep health and behavioral phenotyping. We examined a subset of 164 participants aged 38‐71 with imaging and heart rate data. HRV (std of beat‐to‐beat intervals) was computed from photoplethysmography collected during the neuroimaging procedure. RBA was derived from a machine learning method (Joint and Individual Variation Explained; JIVE) trained using structural features extracted from T1‐weighted MRI data. VO2max was estimated using a modified Balke submaximal testing procedure. RBA, HRV, and VO2max associations were examined using ordinary least squares, controlling for age and sex.

**Result:**

As expected, higher VO2max was associated with younger RBA (Figure 1: t(77) = ‐2.11, p = 0.038) and lower mean HR (t(58) = ‐2.49, p = 0.015). Interestingly, higher HRV (Figure 2: t(161) = ‐1.98, p = 0.049) also predicted younger RBA.

**Conclusion:**

Our findings were conceptually consistent with previous work demonstrating that higher VO2max is associated with greater brain integrity, extending these findings to biological brain aging (RBA). Interestingly, the HRV findings suggest that autonomic integrity may also play a role in slowing apparent brain aging. In combination, these findings suggest that HRV may provide a similar but more accessible window into the physiological factors that influence brain health than VO2max testing. Future work examining the association between autonomic integrity metrics and brain health longitudinally or during interventions may prove fruitful.